# Cross‐Reactive Antibody Responses to Coronaviruses Elicited by SARS‐CoV‐2 Infection or Vaccination

**DOI:** 10.1111/irv.13309

**Published:** 2024-05-09

**Authors:** Richard S. H. Lee, Samuel M. S. Cheng, Jin Zhao, Annie Y. S. Tsoi, Kaman K. M. Lau, CoCo H. C. Chan, John K. C. Li, David S. C. Hui, Malik Peiris, Hui‐Ling Yen

**Affiliations:** ^1^ School of Public Health, LKS Faculty of Medicine The University of Hong Kong Hong Kong China; ^2^ Department of Medicine and Therapeutics CUHK, Prince of Wales Hospital Hong Kong China; ^3^ Centre for Immunology & Infection Hong Kong Science Park Hong Kong China

**Keywords:** convalescent sera, COVID‐19 vaccines, cross‐reactive antibody, human coronaviruses, SARS‐CoV‐2

## Abstract

**Background:**

The newly emerged SARS‐CoV‐2 possesses shared antigenic epitopes with other human coronaviruses. We investigated if COVID‐19 vaccination or SARS‐CoV‐2 infection may boost cross‐reactive antibodies to other human coronaviruses.

**Methods:**

Prevaccination and postvaccination sera from SARS‐CoV‐2 naïve healthy subjects who received three doses of the mRNA vaccine (BioNTech, BNT) or the inactivated vaccine (CoronaVac, CV) were used to monitor the level of cross‐reactive antibodies raised against other human coronaviruses by enzyme‐linked immunosorbent assay. In comparison, convalescent sera from COVID‐19 patients with or without prior vaccination history were also tested. Pseudoparticle neutralization assay was performed to detect neutralization antibody against MERS‐CoV.

**Results:**

Among SARS‐CoV‐2 infection−naïve subjects, BNT or CV significantly increased the anti‐S2 antibodies against Betacoronaviruses (OC43 and MERS‐CoV) but not Alphacoronaviruses (229E). The prevaccination antibody response to the common cold human coronaviruses did not negatively impact the postvaccination antibody response to SARS‐CoV‐2. Cross‐reactive antibodies that binds to the S2 protein of MERS‐CoV were similarly detected from the convalescent sera of COVID‐19 patients with or without vaccination history. However, these anti‐S2 antibodies do not possess neutralizing activity in MERS‐CoV pseudoparticle neutralization tests.

**Conclusions:**

Our results suggest that SARS‐CoV‐2 infection or vaccination may potentially modulate population immune landscape against previously exposed or novel human coronaviruses. The findings have implications for future sero‐epidemiological studies on MERS‐CoV.

## 1 Introduction

SARS‐CoV‐2 is a newly emerged human coronavirus (HCoV) that has rapidly swept through the globe and resulted in significant public health and socioeconomic loss. As a member of the genus Betacoronavirus, SARS‐CoV‐2 possesses shared epitopes with other HCoVs including the common cold Alphacoronaviruses (229E and NL63) and Betacoronaviruses (OC43 and HKU1), as well as two newly emerged Betacoronaviruses, SARS‐CoV‐1 in 2002 and MERS‐CoV in 2012 [1, 2]. Prior exposures to common cold HCoVs may provide cross‐protective humoral or cell‐mediated immunity. However, previous studies showed conflicting results on whether pre‐existing antibodies toward common cold HCoVs provide cross‐protection against SARS‐CoV‐2 infection or severe disease outcomes [3, 4, 5, 6]. On the other hand, pre‐existing memory T cells that are likely elicited in response to common cold CoVs have been consistently associated with cross‐protection among SARS‐CoV‐2‐exposed healthcare workers or household contacts [7, 8].

Since 2020, 7.7 billion SARS‐CoV‐2 infected and re‐infected cases have been reported to WHO and 13.6 billion doses of COVID‐19 vaccines have been administered globally. The extensive exposure to SARS‐CoV‐2 through infection and immunization may substantially affect the population immune landscape and susceptibility to other HCoVs. The pre‐existing immunity to common cold HCoVs may potentially result in back‐boosting of antibodies against the conserved S2 epitopes upon SARS‐CoV‐2 infection or vaccination as reported previously [9, 10, 11, 12, 13]. In addition, de novo antibody response against SARS‐CoV‐2 may also cross‐react with other HCoVs [10, 14]. A better understanding on the cross‐reactive antibody responses may provide guidance on the development of pan‐coronavirus vaccines.

While the spike‐encoding mRNA and the whole‐virion inactivated vaccines have been the main COVID‐19 vaccines administered globally to date, most studies so far have focused on the effect of mRNA vaccines. The mRNA vaccines adopted the prefusion conformation of the spike protein while the prefusion and postfusion conformations have been reported for the inactivated vaccines [15]. Inactivated vaccines also contain additional SARS‐CoV‐2 structural proteins that may stimulate humoral or cell‐mediated immunity. Using prevaccination and postvaccination sera, we firstly compared the cross‐reactive antibody responses against different HCoVs elicited by mRNA and inactivated COVID‐19 vaccines in individuals previously infection‐naïve for SARS‐CoV‐2. We also separately investigated cross‐reactive antibody response in convalescent sera of SARS‐CoV‐2 patients with or without prior vaccination history.

## 2 Methods

### 2.1 Study Design

Sera were collected from healthy subjects enrolled in a longitudinal study for monitoring population immunity to SARS‐CoV‐2 infection and vaccination in Hong Kong. The study was approved by the institutional review board of the Hong Kong West Cluster of the Hospital Authority of Hong Kong (Reference No.: UW20–169) and the Joint Chinese University of Hong Kong‐New Territories East Cluster Clinical Research Ethics Committee (Reference No.: 2020.229). Enrolled participants were followed up every 6 months for sera collection and self‐reported SARS‐CoV‐2 infections that have been RT‐PCR confirmed. Prevaccination and postvaccination sera were collected from age‐matched individuals who received three doses of BioNTech (BNT) (*n* = 20) or three doses of CoronaVac (CV) (*n* = 21) in 2021–2022. The prevaccination sera were collected on the day of receiving the first dose of the BNT or CV vaccine, and the postvaccination sera were collected 4–8 weeks after receiving the third dose of the vaccine.

Due to the “Zero‐COVID” policy adopted in Hong Kong, the majority of the population have remain uninfected until the 5th wave of COVID‐19 outbreak by Omicron BA.2. During this outbreak from January to July 2022, approximately 45% of the Hong Kong population was infected while the previously 4 waves of outbreaks only resulted in <1% cumulative infection attack rate [16]. The convalescent sera were collected from self‐reported participants from January 24, 2022, and March 15, 2022, during the 5th wave of the outbreak, and most of them have acquired SARS‐CoV‐2 infection for the first time. Sera were collected from infected subjects without (*n* = 20) or with vaccination history (*n* = 20 for BNT and *n* = 20 for CV), after self‐reported SARS‐CoV‐2 infections. The archived prepandemic sera in 2019 from healthy blood donors (*n* = 20) were used as controls. The demographic information of the study participants is summarized in Table 1.

**TABLE 1:** Demographics of study participants.

Study participants	Sample size	Age range (median, SD)	Male to female ratio	Mean days postbreakthrough infection (SD)
Postvaccination sera without prior infection history^a^				
Three doses of BNT	20	27–70 (47.5, 12.4)	7:13	Not applicable
Three doses of CV	21	27–73 (49.0, 12.1)	8:13	Not applicable
Convalescent sera^b^				
SARS‐CoV‐2 infection without vaccination	20	21–72 (57.0, 17.6)	7:13	Not applicable
SARS‐CoV‐2 infection plus 2 doses of BNT	10	20–76 (55.0, 17.0)	8:2	Not applicable
SARS‐CoV‐2 infection plus 2 doses of CV	10	48–81 (56.0, 11.6)	6:4	Not applicable
Two doses of BNT followed by SARS‐CoV‐2 infection	10	25–73 (33.0, 14.8)	5:5	32 (13.9)
Two doses of CV followed by SARS‐CoV‐2 infection	10	24–71 (64.0, 14.7)	4:6	41 (7.6)
2019 prepandemic control sera	20	17–56 (31.0, 11.4)	11:18	Not applicable

^a^ The prevaccination sera were collected on the day of receiving the first dose of the BNT or CV vaccine, and the postvaccination sera were collected 4–8 weeks after receiving the third dose of the vaccine. All sera were collected in 2021–2022.

^b^ Convalescent sera were collected from January 24, 2022, and March 15, 2022, during the 5th wave of the outbreak in Hong Kong. Control sera were collected in 2019.

### 2.2 Enzyme‐Linked Immunosorbent Assay (ELISA)

MaxiSorp 96‐well plates (Thermo Fisher) were coated with 0.1 μg recombinant spike [S1, S2, or full‐length S (S1 + S2), as indicated] or nucleocapsid proteins of OC43, 229E, SARS‐CoV‐2, or MERS‐CoV (Sino Biological) per well overnight at 4°C. The plates were washed with PBST (PBS containing 0.05% Tween 20) and blocked with blocking buffer (5% nonfat milk in PBST) for 2 hours. Human sera were heat treated at 56°C for 30 min and were serially threefold diluted from 1:100 to 1:2700 with blocking buffer. Diluted sera (100 μL per well) were added in duplicate to the plate and incubated for 1 hour, followed by detection using 1:10000 diluted HRP‐conjugated goat anti‐human IgG secondary antibody (100 μL per well). TMB substrate (100 μL per well) (Thermo Scientific) was added to the plate for colorimetric signal formation for 10 min and stopped by adding 50 μL per well of 2 M sulphuric acid. Plates were read at wavelength of 450 nm for absorbance (OD 450 nm). In each ELISA plate, the mean OD 450 nm from wells without human sera (*n* = 8 per plate) was calculated as the background. The area under the curve (AUC) was calculated for each serially diluted sera after subtracting the background.

### 2.3 MERS‐CoV Spike Pseudoparticle Neutralization Tests (ppNT)

Luciferase expressing HlV/MERS‐RBD pseudoparticles (5 ng of p24) were preincubated with 1:10 diluted sera at 4°C for 30 min before the mixture was added to Vero E6 cells in triplicates. Infection was determined by quantifying the firefly luciferase activity at 2 days postinfection (Promega Corporation) using the Microbeta luminometer (PerkinElmer). Serum that gave ≥90% reduction of the maximal luciferase activity (e.g., in the absence of antibody) was regarded as the positive. Serum samples were first tested at 1:10 dilution; any sample with a positive signal at 1:10 dilution was further tested to determine the endpoint of inhibition. The highest serum dilution that gave ≥90% reduction of the maximal luciferase activity (e.g., in the absence of antibody) was regarded as the ppNT antibody titre [17].

### 2.4 Statistical Analysis

The difference of grouped AUC of prevaccination and postvaccination against each antigen within vaccination group was analyzed with the Wilcoxon test. The individual AUC difference between prevaccination and postvaccination of the same individual was calculated and compared with the AUC difference between two vaccination groups with the Mann*–*Whitney test. Correlation between AUC ratio of samples against SARS‐CoV‐2 versus other Human Coronaviruses were analyzed using Spearman's rank correlation. The statistical significance of all statistical tests was set at *p* < 0.05.

## 3 Results

### 3.1 COVID‐19 Vaccination Increased Cross‐Reactive Antibody Responses Against Other Betacoronaviruses

We first compared the pre–COVID‐19 and post–COVID‐19 vaccination antibody responses toward various HCoVs among individuals who have not been previously infected with SARS‐CoV‐2. The study population would have been exposed to OC43 and 229E since birth, and SARS‐CoV‐2 exposure may back‐boost antibody response against common epitopes shared with OC43 and 229E. However, the study population should had very limited prior exposure to MERS‐CoV, which would help to ascertain if the cross‐reactive antibody response was *de novo* post–SARS‐CoV‐2 exposure.

**FIGURE 1:**
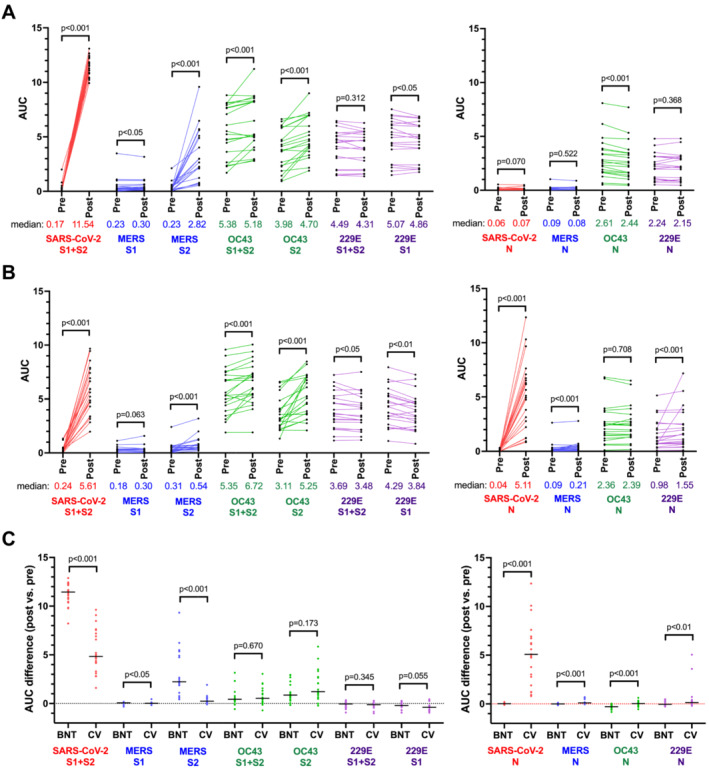
COVID‐19 vaccination increased cross‐reactive antibody responses against other Betacoronaviruses. (A) Prevaccination and postvaccination antibody responses of BNT vaccinees (*n* = 20) to the S and N proteins of different human coronaviruses, Wilcoxon test was used to compare the pre and postvaccination antibody response. (B) Prevaccination and postvaccination antibody responses of CV vaccinees (*n* = 21) to the S and N proteins of different human coronaviruses, Wilcoxon test was used to compare the pre and postvaccination antibody response. (C) Differences between BNT and CV vaccinees in the postvaccination antibody responses against the S and N proteins of different human coronaviruses. Mann*–*Whitney test was used to compare antibody responses of BNT and CV vaccinees.

Both BNT (Figure 1A) and CV (Figure 1B) vaccinees showed significant increase of antibody AUC against the spike protein of Betacoronaviruses after vaccination, including SARS‐CoV‐2 (S1 + S2), MERS‐CoV (S2), and OC43 (S2). In contrast, the postvaccination sera showed a modest, but significant decrease in median antibody AUC against Alphacoronavirus 229E (S1), possible suggesting a waning antibody response over time. Only CV vaccinees showed significantly increased antibody AUC against the nucleocapsid (N) protein of SARS‐CoV‐2, MERS‐CoV and 229E (Figure 1B). Comparing the BNT and CV vaccination responses (Figure 1C), BNT induced significantly greater S‐binding antibody for SARS‐CoV‐2 (S1 + S2) and MERS‐CoV (S2), while CV induced significantly greater N‐binding antibodies for SARS‐CoV‐2, MERS‐CoV, OC43, and 229E.

**FIGURE 2:**
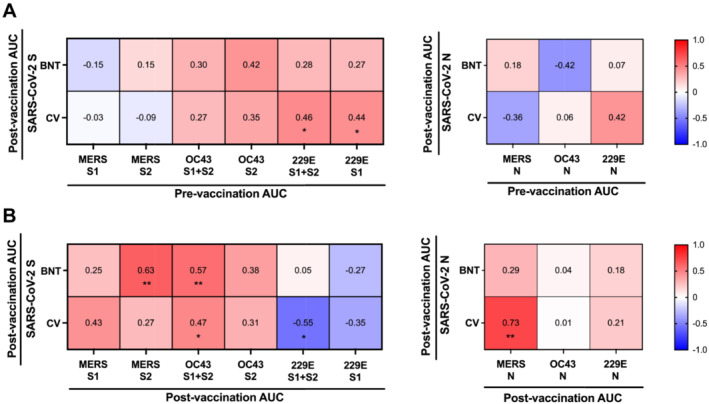
Correlation between postvaccination antibody response against SARS‐CoV‐2 versus antibody response against other human coronaviruses. (A) Postvaccination antibody response against SARS‐CoV‐2 versus prevaccination antibody responses against other HCoVs. (B) Postvaccination antibody response against SARS‐CoV‐2 versus postvaccination antibody responses against other HCoVs. The Spearman coefficient were shown. *p < 0.05, **p < 0.01.

Prevaccination sera showed high baseline binding antibodies for OC43 and 229E (Figure 1A, B). As the baseline antibody titres toward common cold HCoVs may affect the postexposure antibody response against SARS‐CoV‐2 [5], we analyzed the correlation between the prevaccination antibody levels against other HCoVs versus the postvaccination antibody response against SARS‐CoV‐2. For BNT vaccinees, their postvaccination antibody AUC against S of SARS‐CoV‐2 was not affected by the prevaccination antibody against OC43 or 229E (Figure 2A). For CV vaccinees, their postvaccination antibody AUC against S of SARS‐CoV‐2 was marginally correlated with the prevaccination antibody against 229E‐S1 + S2 (Spearman's ρ = 0.46, *p* < 0.05) and 229E‐S2 (Spearman's ρ = 0.44, *p* < 0.05). The postvaccination antibody AUC against the N protein of SARS‐CoV‐2 did not correlate with the prevaccination antibody responses against the N proteins of OC43 or 229E. These results suggest that the de novo antibody response against SARS‐CoV‐2 after vaccination was not negatively affected by the baseline antibody responses against previously exposed HCoV.

The BNT vaccinees' postvaccination antibody AUC against S of SARS‐CoV‐2 was positively correlated with the postvaccination anti‐MERS‐CoV‐S2 (Spearman's ρ = 0.63, *p* < 0.01) and anti‐OC43‐S1 + S2 (Spearman's ρ = 0.57, *p* < 0.01) responses (Figure 2B). For CV vaccinees, the postvaccination antibody AUC against S of SARS‐CoV‐2 was positively correlated with anti‐OC43‐S1 + S2 response (Spearman's ρ = 0.47, *p* < 0.05), and their postvaccination antibody AUC against N of SARS‐CoV‐2 was positively correlated with anti‐MERS‐N response (Spearman's ρ = 0.73, *p* < 0.001) (Figure 2B). Taken together, both the mRNA or inactivated COVID‐19 vaccines may boost antibody responses against conserved epitopes shared with previously exposed (OC43) or novel (MERS‐CoV) Betacoronaviruses.

### 3.2 SARS‐CoV‐2 Infection Increased Cross‐Reactive Antibody Responses Against MERS‐CoV

**FIGURE 3:**
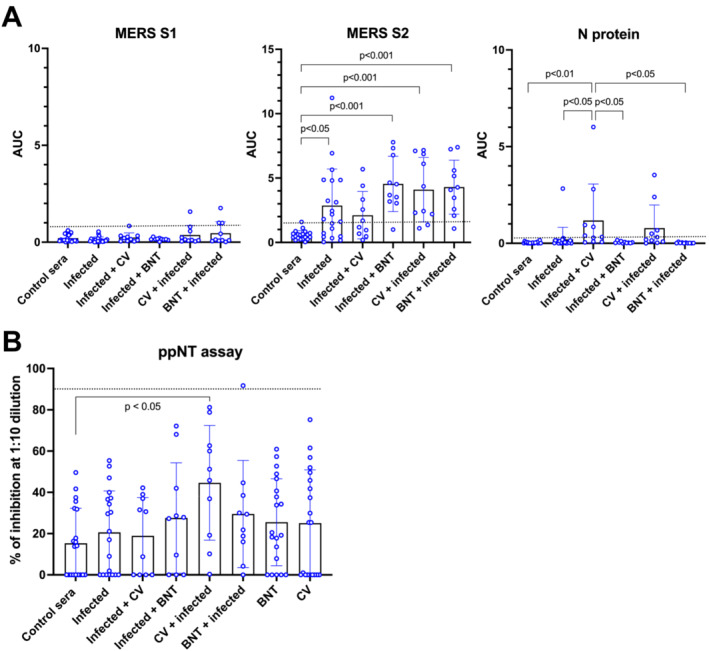
SARS‐CoV‐2 infection increased cross‐reactive antibody responses against MERS‐CoV. (A) Antibody responses against MERS‐CoV S1, S2, or N proteins detected from sera of healthy donors collected in 2019 prior to the COVID‐19 pandemic (control sera, *n* = 20), subjects infected with SARS‐CoV‐2 without vaccination history (infected, *n* = 20), subjects infected with SARS‐CoV‐2 followed by two doses of CV vaccination (infected + CV, *n* = 10), subjects infected with SARS‐CoV‐2 followed by two doses BNT vaccination (infected + BNT, *n* = 10), subjects vaccinated with two doses of CV followed by SARS‐CoV‐2 infection (CV + infected, *n* = 10), subjects vaccinated with two doses of BNT followed by SARS‐CoV‐2 infection (BNT + infected, *n* = 10). The threshold AUC values (mean + 3SD) determined from the prepandemic sera were shown in dotted lines. (B) Neutralizing antibody response against MERS‐CoV were determined using the ppNT assay. In addition to the prepandemic sera and convalescent sera tested above, we also determined neutralizing antibody responses against MERS‐CoV among those who have been vaccinated with three doses of BNT (*n* = 20) or CV (*n* = 20) without a history of SARS‐CoV‐2 infection. Sera were diluted at 1:10 dilution and any sample with ≥90% inhibition was considered positive.

We further investigated if SARS‐CoV‐2 infection may similarly boost cross‐reactive antibody response against MERS‐CoV S and N proteins (Figure 3A). Convalescent sera from SARS‐CoV‐2 patients without a history of COVID‐19 vaccination (*n* = 20), those who have been infected and vaccinated (*n* = 20), and those who were vaccinated and had a breakthrough infection (*n* = 20) were compared with prepandemic sera collected from healthy adults in 2019 (*n* = 20). Compared with the prepandemic sera (using the mean + 3SD AUC value as threshold), 41 out of 60 (71.7%) convalescent sera showed increased antibody against MERS‐CoV S2, while 6 of 60 (10%) showed increased antibody against MERS‐CoV S1. In regard to the antibody response to the S2 protein of MERS‐CoV‐2, those who were vaccinated followed by a breakthrough infection generally showed higher AUC than those who were infected without vaccination history, although the differences were not significant. In addition, those who were infected followed by BNT vaccination showed greater anti‐S2 antibody response than those who were infected followed by CV vaccination, suggesting that BNT vaccination may better expand the breath of antibody response than CV vaccination among those who were infected. Compared with the prepandemic sera, increase in the anti‐N protein antibody AUC was also observed from those who were infected followed by CV vaccination or those who were CV vaccinated followed by breakthrough infection.

### 3.3 Cross‐Reactive Antibody Against MERS‐CoV S2 Were Nonneutralizing

Pseudoparticle neutralization test (ppNT) was used to evaluate if the cross‐reactive anti‐S2 antibodies possess neutralizing activity against MERS‐CoV (Figure 3B). None, except one subject who was vaccinated with BNT followed by SARS‐CoV‐2 infection, showed neutralizing antibody at 1:10 dilution using ppNT assay. Despite being nonneutralizing, sera from subjects with CV vaccination followed by infection (mean ± SD % inhibition = 44.6 ± 27.8) showed greater inhibition against MERS‐CoV than the sera of prepandemic controls (15.3 ± 16.9) (Kruskal–Wallis test, *p* = 0.0435). Taken together, these results suggest that the majority of the cross‐reactive anti‐MERS‐CoV‐S2 antibodies detected after vaccination or infection were nonneutralizing.

## 4 Discussion

Majority of the global population have been exposed to SARS‐CoV‐2 through infection or vaccination to date. As SARS‐CoV‐2 share common epitopes with other HCoVs, it is anticipated that SARS‐CoV‐2 exposure may boost cross‐reactive antibodies toward other HCoV. Using the prevaccination and postvaccination sera of un‐infected subjects, we show that both the mRNA vaccine (BNT) and the inactivated vaccine (CV) increased cross‐reactive antibodies against the S2 protein of the two Betacoronaviruses, OC43 and MERS‐CoV, but not Alphacoronavirus 229E. CV vaccination further boosted anti‐N protein antibodies against MERS‐CoV and 229E. The antibody response against S2 protein of MERS‐CoV were also detected from 41 out of 60 (71.7%) convalescent sera of SARS‐CoV‐2 patients with or without COVID‐19 vaccination history. Our results are in line with a recent study that detected high prevalence of cross‐reactive antibodies to spike proteins of viruses in the *Orthocoronavirinae* among the post–COVID‐19 population [18]. Taken together, these results suggest that SARS‐CoV‐2 exposure may modulate population antibody response toward other human coronaviruses.

The high level pre‐existing antibodies against OC43 and 229E generated from prior infection or vaccination have been shown to impact on the de novo humoral responses against SARS‐CoV‐2 [5]. Among our study subjects, no negative impact was observed in the correlation analysis between prevaccination antibody levels against OC43 or 229E and the postvaccination antibody levels against SARS‐CoV‐2. Due to various public health and social measures implemented in Hong Kong during COVID‐19 pandemic, the activity of various respiratory viruses have been reduced, which may limit recent exposure of our study population to common cold HCoVs. As co‐circulation of SARS‐CoV‐2 and other HCoVs is anticipated, follow up studies are needed to understand how pre‐existing immunity may shape the antibody landscapes of various HCoVs.

Both COVID‐19 vaccines back‐boosted antibodies against the S2 domain of OC43, which aligned with the results reported from previous studies [9, 10, 11, 12, 13]. Furthermore, the de novo antibody response to SARS‐CoV‐2 generated after vaccination or infection were cross‐reactive with S2 of MERS‐CoV. The conserved region on S2 stem‐helix domains across Betacoronaviruses may explain the high cross reactivity of anti‐S2 antibodies [19], while other studies identified cross‐reactive neutralizing antibody that targets S2 region [20, 21, 22, 23]. By comparing the anti‐MERS‐CoV antibodies from infected subjects with or without vaccination history, we noted that those who have been vaccinated, followed by a SARS‐CoV‐2 breakthrough infection generally showed higher anti‐MERS S2 AUC than those who were infected without vaccination history. These study subjects were vaccinated with the prototype virus followed by infection with the BA.2 Omicron variant in 2022. In addition, higher anti‐MERS S2 AUC was detected from those infected followed by BNT vaccination compared with those who were infected without vaccination or those infected followed by CV vaccination. These results suggest that BNT vaccine may stimulate broader antibody response than CV among those who were previously infected. Taken together, these findings have implications for future sero‐epidemiological studies on MERS‐CoV. While binding antibody responses to MERS‐CoV S1 is still likely to be specific for MERS‐CoV infection, binding antibody to MERS‐CoV S2 should no longer be considered as a specific marker for MERS‐CoV infection.

This study is limited by its cross‐sectional study design and each study subject was only assessed at a single time point. However, our study population was unique as the majority of the Hong Kong population have remained uninfected until January 2022 by a large outbreak of Omicron BA.2, which infected around 45% of the population [16]. The postvaccination sera were collected prior to the Omicron BA.2 outbreak, while the convalescent sera were collected from self‐reported participants during the outbreak and most of them have acquired SARS‐CoV‐2 infection for the first time.

The ppNT assay that utilizes pseudovirus that express the full‐length spike would allow detection of neutralizing antibodies targeting the receptor binding domain (RBD) and S2 domain of MERS‐CoV. The results showed that the anti‐MERS‐CoV antibodies were nonneutralizing. Further studies are needed to evaluate if these cross‐reactive antibodies possess Fc‐mediated effector functions and confer protection in vivo.

## Author Contributions

**Richard S. H. Lee:** data curation, formal analysis, investigation, writing–original draft. **Samuel M. S. Cheng:** investigation. **Jin Zhao:** investigation. **Annie Y. S. Tsoi:** investigation. **Kaman K. M. Lau:** investigation. **CoCo H. C. Chan:** investigation. **John K. C. Li:** investigation. **David S. C. Hui:** resources. **Malik Peiris:** formal analysis, funding acquisition, resources, writing–review and editing. **Hui‐Ling Yen:** conceptualization, formal analysis, project administration, supervision, writing–original draft, writing–review and editing.

## Ethics Statement

The study was approved by the institutional review board of the Hong Kong West Cluster of the Hospital Authority of Hong Kong (Reference No. UW20‐169) and the Joint Chinese University of Hong Kong‐New Territories East Cluster Clinical Research Ethics Committee (Reference No. 2020.229).

## Conflicts of Interest

The authors declare no conflicts of interest.

### Peer Review

The peer review history for this article is available at https://www.webofscience.com/api/gateway/wos/peer‐review/10.1111/irv.13309.

## Data Availability

Data are available upon request from the corresponding author.

## References

[1] J. Cui , F. Li , and Z. L. Shi , “Origin and Evolution of Pathogenic Coronaviruses,” Nature Reviews Microbiology 17, no. 3 (2019): 181–192. 30531947 10.1038/s41579-018-0118-9 PMC7097006

[2] N. Kaur , R. Singh , Z. Dar , R. K. Bijarnia , N. Dhingra , and T. Kaur , “Genetic Comparison Among Various Coronavirus Strains for the Identification of Potential Vaccine Targets of SARS‐CoV2,” Infection, Genetics and Evolution 89 (2021): 104490. 10.1016/j.meegid.2020.104490 PMC7395230 32745811

[3] K. W. Ng , N. Faulkner , G. H. Cornish , et al., “Preexisting and De Novo Humoral Immunity to SARS‐CoV‐2 in Humans,” Science 370, no. 6522 (2020): 1339–1343. 33159009 10.1126/science.abe1107 PMC7857411

[4] E. M. Anderson , E. C. Goodwin , A. Verma , et al., “Seasonal Human Coronavirus Antibodies Are Boosted Upon SARS‐CoV‐2 Infection but Not Associated With Protection,” Cell 184, no. 7 (2021): 1858–64 e10. 33631096 10.1016/j.cell.2021.02.010 PMC7871851

[5] C. Y. Lin , J. Wolf , D. C. Brice , et al., “Pre‐Existing Humoral Immunity to Human Common Cold Coronaviruses Negatively Impacts the Protective SARS‐CoV‐2 Antibody Response,” Cell Host & Microbe 30, no. 1 (2022): 83–96 e4. 34965382 10.1016/j.chom.2021.12.005 PMC8648673

[6] P. R. Wratil , N. A. Schmacke , B. Karakoc , et al., “Evidence for Increased SARS‐CoV‐2 Susceptibility and COVID‐19 Severity Related to Pre‐Existing Immunity to Seasonal Coronaviruses,” Cell Reports 37, no. 13 (2021): 110169. 34932974 10.1016/j.celrep.2021.110169 PMC8648802

[7] L. Swadling , M. O. Diniz , N. M. Schmidt , et al., “Pre‐Existing Polymerase‐Specific T Cells Expand in Abortive Seronegative SARS‐CoV‐2,” Nature 601, no. 7891 (2022): 110–117. 34758478 10.1038/s41586-021-04186-8 PMC8732273

[8] R. Kundu , J. S. Narean , L. Wang , et al., “Cross‐Reactive Memory T Cells Associate With Protection Against SARS‐CoV‐2 Infection in COVID‐19 Contacts,” Nature Communications 13, no. 1 (2022): 80. 10.1038/s41467-021-27674-x PMC8748880 35013199

[9] E. A. Elko , G. A. Nelson , H. L. Mead , et al., “COVID‐19 Vaccination Elicits an Evolving, Cross‐Reactive Antibody Response to Epitopes Conserved With Endemic Coronavirus Spike Proteins,” Cell Reports 40, no. 1 (2022): 111022. 35753310 10.1016/j.celrep.2022.111022 PMC9188999

[10] E. S. Geanes , C. LeMaster , E. R. Fraley , et al., “Cross‐Reactive Antibodies Elicited to Conserved Epitopes on SARS‐CoV‐2 Spike Protein After Infection and Vaccination,” Scientific Reports 12, no. 1 (2022): 6496. 35444221 10.1038/s41598-022-10230-y PMC9019795

[11] A. R. Crowley , H. Natarajan , A. P. Hederman , et al., “Boosting of Cross‐Reactive Antibodies to Endemic Coronaviruses by SARS‐CoV‐2 Infection but Not Vaccination With Stabilized Spike,” eLife 11 (2022): 11. 10.7554/eLife.75228 PMC8923670 35289271

[12] F. Amanat , M. Thapa , T. Lei , et al., “SARS‐CoV‐2 mRNA Vaccination Induces Functionally Diverse Antibodies to NTD, RBD, and S2,” Cell 184, no. 15 (2021): 3936–3948.e10. 34192529 10.1016/j.cell.2021.06.005 PMC8185186

[13] T. Aydillo , A. Rombauts , D. Stadlbauer , et al., “Immunological Imprinting of the Antibody Response in COVID‐19 Patients,” Nature Communications 12, no. 1 (2021): 3781. 10.1038/s41467-021-23977-1 PMC8213790 34145263

[14] S. M. Murray , A. M. Ansari , J. Frater , et al., “The Impact of Pre‐Existing Cross‐Reactive Immunity on SARS‐CoV‐2 Infection and Vaccine Responses,” Nature Reviews Immunology 23, no. 5 (2023): 304–316. 10.1038/s41577-022-00809-x PMC9765363 36539527

[15] F. X. Heinz and K. Stiasny , “Distinguishing Features of Current COVID‐19 Vaccines: Knowns and Unknowns of Antigen Presentation and Modes of Action,” Npj Vaccines 6, no. 1 (2021): 104. 34400651 10.1038/s41541-021-00369-6 PMC8368295

[16] J. J. Lau , S. M. S. Cheng , K. Leung , et al., “Real‐World COVID‐19 Vaccine Effectiveness against the Omicron BA.2 Variant in a SARS‐CoV‐2 Infection‐Naive Population,” Nature Medicine 29, no. 2 (2023): 348–357. 10.1038/s41591-023-02219-5 PMC9941049 36652990

[17] R. A. Perera , P. Wang , M. R. Gomaa , et al., “Seroepidemiology for MERS Coronavirus Using Microneutralisation and Pseudoparticle Virus Neutralisation Assays Reveal a High Prevalence of Antibody in Dromedary Camels in Egypt, June 2013,” Euro Surveillance 18, no. 36 (2013): pii=20574. 24079378 10.2807/1560-7917.es2013.18.36.20574

[18] G. Singh , A. Abbad , G. Kleiner , et al., “The Post‐COVID‐19 Population Has a High Prevalence of Cross‐Reactive Antibodies to Spikes From All Orthocoronavirinae Genera,” MBio 15 (2023): e0225023. 38112467 10.1128/mbio.02250-23 PMC10790767

[19] M. M. Sauer , M. A. Tortorici , Y. J. Park , et al., “Structural Basis for Broad Coronavirus Neutralization,” Nature Structural & Molecular Biology 28, no. 6 (2021): 478–486. 10.1038/s41594-021-00596-4 33981021

[20] C. Dacon , C. Tucker , L. Peng , et al., “Broadly Neutralizing Antibodies Target the Coronavirus Fusion Peptide,” Science 377, no. 6607 (2022): 728–735. 35857439 10.1126/science.abq3773 PMC9348754

[21] G. Song , W. T. He , S. Callaghan , et al., “Cross‐Reactive Serum and Memory B‐Cell Responses to Spike Protein in SARS‐CoV‐2 and Endemic Coronavirus Infection,” Nature Communications 12, no. 1 (2021): 2938. 10.1038/s41467-021-23074-3 PMC8134462 34011939

[22] P. Zhou , G. Song , H. Liu , et al., “Broadly Neutralizing Anti‐S2 Antibodies Protect Against All Three Human Betacoronaviruses That Cause Deadly Disease,” Immunity 56, no. 3 (2023): 669–86 e7. 36889306 10.1016/j.immuni.2023.02.005 PMC9933850

[23] D. Pinto , M. M. Sauer , N. Czudnochowski , et al., “Broad Betacoronavirus Neutralization by a Stem Helix‐Specific Human Antibody,” Science 373, no. 6559 (2021): 1109–1116. 34344823 10.1126/science.abj3321 PMC9268357

